# General practitioner obstetricians’ models of care in rural Western Australia

**DOI:** 10.1111/ajo.13483

**Published:** 2022-01-22

**Authors:** Maryam Wazir, Carly Roxburgh, Sarah Moore

**Affiliations:** ^1^ 2720 Rural Clinical School of WA University of Western Australia Bunbury Australia; ^2^ Rural Clinical School of WA University of Western Australia Albany Australia; ^3^ 2720 Rural Clinical School of WA University of Western Australia Busselton Australia

**Keywords:** rural health, maternal health services, workforce, rural generalism

## Abstract

**Background:**

In Australia, a significant proportion of women live rurally and deliver their babies in services supported by general practitioner obstetricians (GPOs). While GPOs are known to be an important backbone in the provision of maternity care in Australia, little attention has been paid to their models of care.

**Aims:**

To describe the models of maternity care provided by GPOs across Western Australia.

**Materials and Methods:**

This was a multi‐phase mixed‐methods cross‐sectional exploratory study. We invited rural GPOs in Western Australia to complete an online survey about their models of care and a sub‐group of GPOs agreed to an interview to further explore their responses.

**Results:**

Thirty‐five GPOs completed the survey and 12 completed an interview. We found that GPOs work in a variety of models, dependent on local community needs, resources and geography. Key attributes of GPO models are continuity of care, safety, generalism, accessibility and affordability. GPO care involves continuity of care beyond the time limits of pregnancy.

**Conclusions:**

GPOs’ models of care make up an essential part of rural maternity services and have evolved to meet the needs of the communities they serve. This work informs rural generalist trainees of career pathways and policymakers about rural service provision.

## INTRODUCTION

Western Australia (WA) covers a vast land area of 2.6 million square kilometres with a population of 2.6 million people. Of these, 546 000 (21%) live in rural and remote areas. The state’s only tertiary obstetric referral centre is located in Perth. Annually 4500 of 34 000 births in WA are in regional locations, with maternity care provided at 18 public hospitals and two private hospitals in rural and remote WA towns. Obstetric care is provided by general practitioner obstetricians (GPOs) in all 18 rural maternity units, with on‐site support from obstetrician‐gynaecologists, paediatricians and anaesthetists in only six regional hospitals.^1^ GPOs have dual skills in general practice (usually by Fellowship of either the Royal Australian College of General Practitioners or Australian College of Remote and Rural Medicine) and in obstetrics, usually by Diploma (DRANZCOG) or Advanced Diploma (DRANZCOG‐Adv) of the Royal Australian and New Zealand College of Obstetricians and Gynaecologists (RANZCOG). Currently, there are 2070 basic diplomates and 561 advanced diplomates registered with RANZCOG, representing two‐thirds of college membership.^2^ Rural Health West, the rural workforce agency in WA, estimates that there are 94 GPOs and 16 obstetrician‐gynaecologists working in rural WA.^3^

Historically, training pathways for GPOs have been uncoordinated and self‐directed, relying on personal relationships with mentors and training hospitals. The National Rural Generalist Pathway has an explicit goal of training more rural procedural GPs,^4^ but we note that there is little information publicly available regarding GPO as a model of maternity care for women or as a career path. Australia‐wide, there is a trend toward promoting midwifery‐led care, on the basis that this will reduce interventions such as caesarean sections, instrumental deliveries and epidurals, while increasing maternal satisfaction. There has been little academic attention paid to the role of GPs or GPOs on outcomes in maternity care. A recent Queensland‐based study demonstrated that GPO‐supported birthing units had strong indicators of quality and safety.^5^

The National Rural Generalist Taskforce recognise the importance of supporting and valuing of rural generalists, including GPOs. Their role is unique and requires high‐quality, networked training, career structure and recognition, remuneration and professional supports in order to retain rural generalists in the country and attract the next generation of doctors to this field.^4^ The WA Country Health Service (WACHS) Maternal and Newborn Care Strategy identifies GPOs as important members of maternity services in order to deliver women‐centred care and highlights the need for GPO training pathways and multidisciplinary training activities that ensure GPOs can develop and maintain their professional skills.^1^

The Australian Maternity Care Classification System refers to ‘GPO care’ as a single model of care akin to a private obstetrics model: ‘Antenatal care is provided by a GPO. Intrapartum care is provided in a private or public hospital by the GPO and hospital midwives. Postnatal care is provided in the hospital by the GPO and hospital midwives and may continue in the home or community.’^6^ We hypothesised that, due to the wide variation in service size, support and staffing expertise across the state, a variety of different models of care are provided by GPOs across rural and remote WA. The aims of this observational study were to describe the models of maternity care actually provided by GPOs, and to identify perceived strengths and stressors on the model.

## METHODS AND MATERIALS

### Study design

A multi‐phased mixed‐methods cross‐sectional exploratory study was conducted with an initial online survey followed by a series of qualitative semi‐structured face‐to‐face or videocall interviews.

### Participants

Participants were doctors working in WA who identified as GPOs and who subscribed to the WA GPO Network email communication group. The group includes 82 of an estimated 94 GPOs in WA.^2^

### Materials

The online survey was conducted on RedCap^©^ software^7^ and included demographics, models of care and some open questions about the models of care. Participants were invited to participate in an interview at the end of the survey. Demographic data were analysed with descriptive statistics including frequencies and cross‐tabulation using Microsoft Excel^©^. Interviews were transcribed using a professional transcription service. Qualitative data were analysed by the study authors using thematic analysis.^8^

### Ethics approval

Ethics approval was obtained from the University of Western Australia Human Research Ethics Committee (RA/4/20/4998).

## RESULTS

Thirty‐five of 82 GPOs completed the online survey. They were located in 15 different towns, ranging between 70 and 3000 km from Perth (Mandurah and Kununurra respectively), encompassing the whole state which includes some of the most isolated locations in the world. While 25 participants consented to interview, saturation was reached after 12 interviews with no new perspectives being raised by participants.

GPO demographic information is presented in Table 1. The age range of participants was 29–58 (median 39) years and duration of practice as a GPO ranged from 1–35 (median eight) years.

**Table 1:** Demographics of participating GPOs

	Number of GPOs (*n* = 35)	%
Age		
25–30	6	17.1
31–40	14	40.0
41–50	6	17.1
51–60	7	20.0
61–64	2	5.7
Gender		
Male	14	40
Female	21	60
Qualifications		
Basic DRANZCOG	15	42.8
Advanced DRANZCOG	20	57.1
Years of practice as GPO		
0–5	15	42.8
6–10	8	22.9
11–15	0	0
16–20	6	17.1
>20	6	17.1
Location of GPO work (MMM)		
MMM1	1	2.8
MMM2	5	14.3
MMM3	15	42.9
MMM4	2	5.7
MMM5	2	5.7
MMM6	6	17.1
MMM7	4	11.4
Hospital‐based services		
Public	15	42.8
Public and private	18	51.4
None	2	5.7
Hospital employment status		
Salaried (SMP, DMO)	8	22.9
Fee‐for‐service (VMP)	23	65.7
Locum GPO	2	5.7
NA	2	5.7
On‐call days per week (median 4)		
0	2	5.7
1	4	11.4
2	11	31.4
3	5	14.2
4	3	8.6
5	3	8.6
6	2	5.7
7	5	14.2
Percentage of practice that is maternity care		
0–20%	13	37.1
21–40%	10	28.6
41–60%	5	14.3
61–80%	4	11.4
81–100%	3	8.6
Number of births personally managed per year		
0–20	8	22.9
21–40	5	14.3
41–60	6	17.1
61–80	4	11.4
81–100	6	17.1
101–120	3	8.6
121–140	1	2.9
>140	3	8.6

DMO, District Medical Officer; DRANZCOG, Royal Australian and New Zealand College of Obstetricians and Gynaecologists Diploma; GPO, general practitioner obstetrician; MMM, Modified Monash Model Category; SMP, Senior Medical Practitioner; VMP, Visiting Medical Practitioner.

Geographical locations of participants ranged from Modified Monash Model (MMM) categories 1–7, representing all levels of rurality from metropolitan to very remote communities.^9^ GPOs reported that the number of GPOs in their town varied from two to ten, with more GPOs in larger rural towns in WA. Fourteen GPOs worked in locations without a specialist obstetrician‐gynaecologist on site. All GPOs had either DRANZCOG or DRANZCOG‐Adv qualifications, and so represented typical GPOs registered with RANZCOG in Australia. The number of on‐call days per week ranged from one to seven and the median was four days. There was a wide variation in the number of deliveries personally managed per year, ranging from zero (GPOs providing shared care only) to 360 (a GPO with a mostly private practice).

### Scope of practice

In addition to low‐risk intrapartum care, 27 (77%) surveyed GPOs independently perform vacuum deliveries, 15 (43%) perform forceps deliveries and 17 (49%) perform caesarean sections. Table 2 shows procedures self‐reported as being undertaken by GPOs. Twenty‐one GPOs (60%) have on‐site support from local specialist obstetrician‐gynaecologists for surgical management of complex cases. One hundred percent of surveyed GPOs reported regularly attending education and training courses, including neonatal resuscitation, cardiotocography interpretation and obstetric emergencies, which are necessary to maintain credentialing to practice.

**Table 2:** Self‐reported procedures undertaken by GPOs

Procedures	Number of GPOs (*n* = 35) performing procedures	
	Independently	Under supervision
Vacuum delivery	27 (77%)	3 (9%)
Forceps delivery	15 (43%)	11 (31%)
Caesarean section	17 (49%)	1 (3%)
Operative management of postpartum haemorrhage	19 (54%)	5 (14%)
3rd degree perineal tear repair	13 (37%)	11 (31%)
4th degree perineal tear repair	4 (11%)	12 (34%)
Surgical ectopic pregnancy management	0 (0%)	15 (43%)

GPO, general practitioner obstetrician

### Qualitative findings: models of care

GPOs in this study reported providing care that included the whole continuum of pregnancy from preconception, to antenatal, intrapartum and postpartum care. They also provide routine general practice care, including management of comorbid medical problems and mental health care. GPOs reported working both independently and collaboratively. The other maternity care providers they collaborated with – specialist obstetricians, midwives, GPs and allied health providers – focused on midwives, with whom they most commonly worked in hospital‐based midwifery care. Regional specialist obstetricians were cited as providing support to GPOs caring for high‐risk women; however, as only a minority of WACHS hospitals have obstetricians, GPOs are sometimes required to independently manage high‐risk situations. In this instance, GPOs reported using regional or tertiary‐based phone support. GPOs from eight hospitals reported they had no local paediatricians, so GPOs provided routine and emergency neonatal care, again supported remotely by regional paediatricians and the WA Newborn Emergency Transport Service (NETS WA). GPOs were familiar with their local hospital arrangements for referral pathways and emergency backup.

GPOs who worked in larger towns reported a choice of maternity models, including GPO shared care with midwives (as above), plus independent midwifery programs (three towns) with GPO backup, as well as private obstetricians (two regional centres). GPOs reported that the majority of regional women would need to travel away from home to access these models. It was evident in surveys and in interview that GPOs are keenly aware of collaborating with many other healthcare providers such as GP anaesthetists, GPs, nurses, theatre staff, Aboriginal health workers, psychologists, women’s health physiotherapists, lactation consultants, social workers, dieticians, diabetes educators and exercise physiologists when available in a region.

There was a distinct difference between employment arrangements between models that were divided by the 26th parallel. In southern sites, GPOs’ reports were predominantly about providing antenatal and postnatal care in private practice or Aboriginal Medical Service setting, with intrapartum care in public or private hospitals under a visiting medical practitioner (fee‐for‐service) arrangement. GPO on‐call rosters varied from town to town depending on needs of the GPOs and the community. Some GPOs were on‐call for their women seven days a week while other towns reported a shared roster. In northern sites, the convention was to salary GPOs who then provided rostered services to hospital‐based antenatal clinics, labour wards and inpatients. In addition to this workload some additionally chose to work in private general practice, remote clinics or Aboriginal Medical Services outside the hospital.

Strengths of the GPO model of care are summarised with illustrative quotations in Table 3 and include continuity of care; holistic care; cultural safety; job satisfaction; safety and generalism.

**Table 3:** Strengths of general practitioner obstetrician model of care

1. Continuity of care	‘We end up building quite a good relationship with these patients. And so, if you do have an adverse outcome where you end up in theatre, they have built that trust and they trust that when you say “look, we need to intervene”. And it may not have been with their plan but, because you’ve had that continuity, they are a lot more open and understanding and I think they cope a lot better with that.’ Participant D
2. Holistic care	‘I saw them when they were trying to get pregnant and I saw them when they were having a baby and then I see them after and I see their kids after. I look after all their GP matters as well.’ Participant F
3. Cultural safety	‘Our Aboriginal Medical Service has a very holistic approach to patient care. It’s a walk‐in service and we’ve improved access to other services there over time. We have a family centre, we only look after women and children, we don’t see any men there.’ Participant C
4. Job satisfaction	‘Women value continuity of care with their carer. I think it’s safer and far more satisfying for the doctor. If you actually have a relationship with a patient, there’s always something interesting about them.’ Participant A
5. Safety	‘We do have quite a safe model here where we have specialist O&G providing opinions, we have a good midwifery and GP obstetricians team and we have access to theatres and GP anaesthetists.’ Participant C
6. Generalism	‘You need to have someone who’s prepared to repair a simple episiotomy, but also prepared to do a difficult C‐section. And the neonatal resuscitation afterwards. It’s just the limitation of numbers of populations versus the skills that are needed. So in a place like here, generalism is the thing that will work.’ Participant L

The stressors on the GPO model of care related to local workforce factors, patient needs and access to care, and recognition of GPO as a career and speciality (see Table 4). Local factors included the closure of smaller maternity units and restriction of services such as theatre closure meaning women needed to be transferred out for caesarean section. Some GPOs are actively working to attempt reinstatement of caesarean section services, but have not yet been successful. Some GPOs report being suddenly excluded from local hospitals through changes to local models of care.

**Table 4:** Stressors on GPO model of care

1. Local workforce sustainability	‘If I leave now, there will be a lack of full‐time obstetrics cover.’ Participant G
2. Onerous on‐call roster	‘Our GPOs have their own patients apart from on weekends, where we have a semi‐formal roster system and we take it in turns to cover the weekend. Having said that, the GPOs often need to call in an extra person, for example if you have to do a caesarean, you need an assistant and a doctor to do the neonatal resuscitation. It’s onerous to always be on‐call and younger GPOs are less willing to do it.’ Participant A
3. Inadequate resources to meet patient needs	‘Accommodation is a problem. It’s one of the reasons that patients can be resistant to being transferred. The PATS [patient assisted transport service] officers do a great job, but it is difficult if people suddenly get a complication, moving them, not being able to tell them where they will stay and what happens and just asking them to have faith in the system. Their partners and children aren’t covered by PATS and they will often take substantial obstetric risks in order to deliver locally. Like a woman who has four children and who lives remotely and who doesn’t trust her family not to be drunk while they are caring for her children is going to put her pregnancy at risk to protect her existing children rather than being transferred.’ Participant K ‘I do think it’s pretty important that women have the option of delivering close to home. If we have to send someone out, we make sure she understands why, but then we try to negotiate, so that as soon as she’s had her baby, if all goes well, she can come back here to the local hospital.’ Participant C
4. Patient access to other services such as radiology	‘The main thing I think we need to do is improve access to ultrasound services. So we’ve got a private radiology – the hospital runs radiology but the hospital’s radiology department is overwhelmed with scans, so the wait list is often quite long. It can be up to six weeks or so’ Participant B
5. Recognition of GPO skills	‘Whilst the label here is a low‐risk service, the reality is it’s high risk, because of the population and geography, and the health service needs to recognise that and staff it appropriately. Every year there are examples of very high‐risk obstetrics’ Participant E

GPO, general practitioner obstetrician.

## DISCUSSION

To our knowledge, this is the first study to describe actual models of GPO care being practised in WA, based on a large sample of currently practising GPOs. We argue that this large and representative sample of GPOs from across the state have a demonstrated role in providing extensive care to women, throughout preconception, antenatal, intrapartum and postnatal care, as well as longitudinal care across generations. We found that on‐call and scope‐of‐practice arrangements were diverse and adapted to local communities’ hospital arrangements, requirements and practice capabilities. Some services were embedded in community general practice or Aboriginal Medical Services while others were based exclusively in hospitals. GPOs worked collaboratively with members of the maternity healthcare team, particularly midwives, obstetrician‐gynaecologists and paediatricians. In rural and remote settings it is particularly important that all members of the maternity team can work closely and communicate effectively, as we describe.

Local arrangements for collaboration, referral and backup differed markedly depending on a the presence of a critical mass of GPOs, GP anaesthetists, midwives and theatre staff – a critical mass that has been eroded resulting in the closure of rural maternity units across Australia.^10^ Recently efforts have been made to halt this trend, and so to enable more rural women to deliver locally, surrounded by family.^4^

In contrast some larger towns have shown a swing toward GPOs being excluded from intrapartum care as models move to obstetrician‐gynaecologist‐led care supported by midwives. We observe that there is a delicate balance between too many and too few obstetric providers: enough to provide a service and avoid burn‐out due to excessive on‐call demands, while conversely oversupply results in dilution of opportunities to maintain skills such as caesarean section or neonatal resuscitation. Deliberate planning of GPO services is necessary for career stability and to demonstrate a clear career path to aspiring trainees. It is critical to engage GPOs in service development, monitoring and management of maternity services to achieve high‐quality maternity services. In WA, the importance of active involvement of GPOs in clinical governance of maternity units in order to maintain safe and appropriate maternity services in the country has been recognised.^1^

The funding mechanisms for rural maternity care vary geographically in WA. In the southern regions, where GPOs provide antenatal and postnatal care in private general practice, funding is through Medicare (federal funding) and private fees. Hospital‐based outpatient clinics, run by GPOs in the northern regions, are funded through state‐run WACHS. Throughout WA inpatient maternity services are funded by the state, apart from a small number of services claimed from private health funds. This shifting of costs between state and federally funded health budgets needs to be considered in service design and planning.

Continuity of care has been identified as a key element of maternity care as it builds positive relationships, enhances communication and enables timely access to relevant information between women, their families and the healthcare team.^1^ Continuity of care is a well‐known strength of midwifery models of care.^11^ We found that continuity is a key characteristic of GPO care. In addition to benefits for women, continuity of care, often extending far beyond the perinatal period, was a common source of job satisfaction for the surveyed GPOs.

Unlike historical GPO workforce demographics, respondents to our study were 60% female, youthful, and many relatively new to practising as a GPO (less than five years experience).^12^ This creates unique workforce challenges including flexible training, rostering, mentoring and skills maintenance.

### Strengths and limitations

The strength of this study is that we collected data from GPOs from 15 of 18 rural towns in Western Australia where GPO services are provided, offering a strongly representative sample of care models that are actually being practised in rural WA. A source of possible bias is that the two supervising authors are currently practising GPOs in rural WA. We collected and analysed the data using thematic analysis, an established qualitative method, to minimise any bias that this could introduce. This study invites investigating models of GPO care in other states of Australia. Further research should also include mapping of maternity care services, including the distribution of workforce, levels of service provision stratified by risk, as well as consumer perspectives on what is most important to them as this may differ markedly from clinicians’ perspectives.

In summary, this study offers an initial insight into current real‐life GPO models of care in rural WA. It demonstrates the essential role GPOs play in rural maternity services in WA, meeting local community needs, as well as providing collaborative working relationships between GPOs, midwives, specialists and other healthcare professionals. It is already known that GPOs in Australia are trained to provide high standards of obstetric and neonatal care, and that their presence in rural maternity units ensures women can access safe and person‐centred maternity care close to home. In conclusion, we highlight that rural maternity services face the challenge of maintaining GPO workforce numbers and skills; however, with proactive planning and support, we suggest that GPO models of care have the solidarity to be able to consolidate and develop further as needed. We argue that GPOs should be recognised for the service they provide toward making strong communities, and should be actively included in maternity service planning and policy.

## Funding

Open access publishing facilitated by The University of Western Australia, as part of the Wiley ‐ The University of Western Australia agreement via the Council of Australian University Librarians.
